# Characterization of a Novel Nanocomposite Film Based on Functionalized Chitosan–Pt–Fe_3_O_4_ Hybrid Nanoparticles

**DOI:** 10.3390/nano11051275

**Published:** 2021-05-13

**Authors:** Sangeeta Kumari, Raj Pal Singh, Nayaku N. Chavan, Shivendra V. Sahi, Nilesh Sharma

**Affiliations:** 1 Department of Biological Sciences, University of the Science, Philadelphia, PA 19104-4495, USA; s.sahi@usciences.edu; 2 Advanced Research Centre in Pharmaceutical Sciences & Applied Chemistry, Bharati Vidyapeeth University, Erandawane, Pune 411038, India; rp.singhncl@gmail.com; 3 Division of Polymer Science and Engineering, CSIR-National Chemical Laboratory, Dr. Homi Bhabha Road, Pune 411008, India; nn.chavan2007@gmail.com; 4 Department of Biology, Western Kentucky University, 1906 College Heights Boulevard, Bowling Green, KY 42101-1080, USA; nilesh.sharma@wku.edu

**Keywords:** chitosan, Pt–Fe_3_O_4_ hybrid nanoparticles, thermogravimetric analysis, nanocomposite films, tensile strength testing

## Abstract

The development of organic—inorganic hybrids or nanocomposite films is increasingly becoming attractive in light of their emerging applications. This research focuses on the formation of a unique nanocomposite film with enhanced elasticity suitable for many biomedical applications. The physical property measurement system and transmission electron microscopy were used to analyze Pt–Fe_3_O_4_ hybrid nanoparticles. These nanohybrids exhibited magnetic effects. They were further exploited to prepare the nanocomposite films in conjunction with a chitosan-g–glycolic acid organic fraction. The nanocomposite films were then examined using standard techniques: thermogravimetric analysis, X-ray diffraction, Fourier transform infrared spectroscopy, and atomic force microscopy. Tensile strength testing demonstrated a significantly greater elastic strength of these nanocomposite films than pure chitosan films. The water absorption behavior of the nanocomposites was evaluated by measuring swelling degree. These nanocomposites were observed to have substantially improved physical properties. Such novel nanocomposites can be extended to various biomedical applications, which include drug delivery and tissue engineering.

## 1. Introduction

Multifunctional hybrid materials have become more prevalent [1] and polymer–nanoparticles nanocomposites have earned accelerated interest in material science and biological applications as a result of their efficacious properties derived from a variety of constituents. The combined properties of organic polymers and inorganic nanoparticles, such as flexibility, magnetic, mechanical [2], thermal [3], electrical [4], and optical properties [5], were observed in these nanocomposites. This enhancement depends upon the incorporation of nanoparticles and their state of dispersion in the polymer matrix [6,7]. Nanoparticles disperse in a chitosan matrix very efficiently. Its interfacial adhesion eliminates scattering and allows the exciting possibility of developing membranes, coatings, and elastic films.

Nanocomposites of polymer–magnetic (Fe_3_O_4_) nanoparticles are widely applied in the biomedical field, such as in drug delivery applications [8,9] and the treatment of cancer cells by hyperthermia [10,11]. Various types of multicomponent nanoparticles have been reported so far, such as Au–Fe_3_O_4_ hybrid nanoparticles and Co_3_O_4_–Fe_3_O_4_ hybrid nanoparticles, which are synthesized by chemical methods. These hybrid nanoparticles are used along with grafted chitosan for making scaffolds, which are used in drug delivery studies [12,13]. It is reported prominently that these hybrid nanostructures, when linked with components, enhance the physical, as well as the chemical, properties of the components. Several other approaches for synthesizing multicomponent nanoparticles have also been reported [14,15,16,17,18,19,20,21]. Here, we have discussed a procedure for synthesizing Pt–Fe_3_O_4_ hybrid nanoparticles, which differ in respective size and number of iron oxide particles. This method is an extension of the original procedure reported by Sun et al. [20], which involves thermal degradation of Fe(CO)_5_. Chitosan is known to be the second most abundant natural polymer. It is a β−(1,4)-linked D-glucosamine derivative of chitin, and the natural polysaccharide with the second greatest abundance. It is also a well-known efficient chelating agent for metals and proteins [22], making it efficient in the production of sensors [23]. The amino group (pKa 6.2) makes it an excellent processible polymer. The chitosan polymer has reactive functional groups such as amino and hydroxyl, which facilitate the surface modification of the chitosan. This surface modification by grafting on to chitosan induces significant structural changes [24,25]. This paper reports a novel high-quality, self-sustained film consisting of biopolymeric glycolic acid-grafted chitosan matrices containing Pt–Fe_3_O_4_ hybrid nanoparticles. It is expected that the grafted chitosan chain can act as an internal plasticizer to soften and elasticize chitosan films by reducing their brittleness. These nanocomposite films exhibit interesting elastic properties and could be used in multiple biomedical applications such as drug delivery and tissue engineering [26,27,28]. 

## 2. Materials and Methods

Chitosan (Mw = 1.5 × 10^5^) with 85% degree of deacetylation, 99% purified glycolic acid, chloroplatinic acid hexahydrate (HPtCl_6_·6H_2_O), 1-octadecene, iron pentacarbonyl (Fe(CO)_5_), oleic acid (OA), and oleylamine (OAM) were bought from Sigma Aldrich (St. Louis, MO, USA). Sisco Research Laboratories Pvt. Ltd., (Mumbai, India) provided the phenyl ether. Ultra-pure water was used throughout the work.

### 2.1. Pt–Fe_3_O_4_ Hybrid Nanoparticle Synthesis

**Scheme 1:**
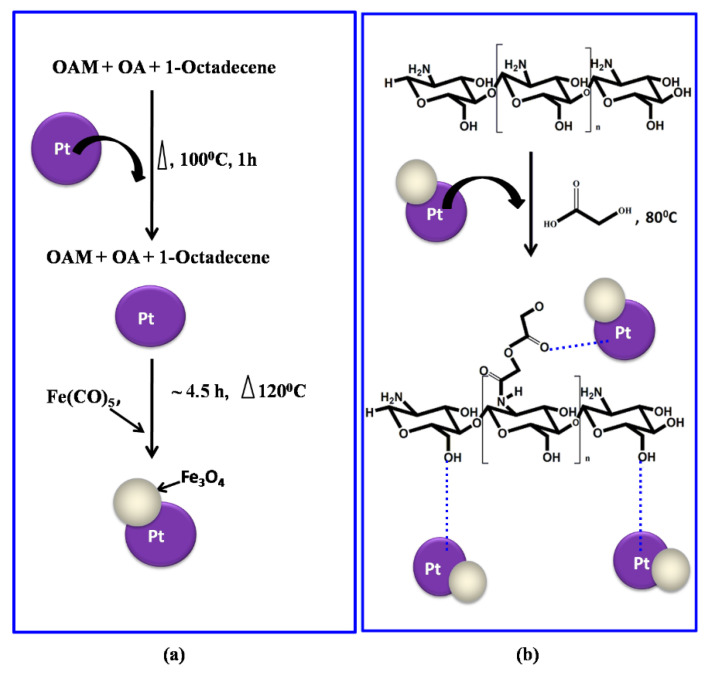
(**a**) Synthesis of Pt–Fe_3_O_4_ hybrid nanoparticles, (**b**) grafted chitosan (GA-g–CS) synthesis and synthesis of GA-g–CS–Pt–Fe_3_O_4_ nanoparticles nanocomposites.

H_2_PtCl_6_.6H_2_O undergoes thermal decomposition at high temperatures to produce platinum nanoparticles (PtNP). The reacting component contained 1,2-tetradecanediol, phenyl ether, H_2_PtCl_6_·6H_2_O, and OAM was heated to 185 °C under an inert condition. Furthermore, ethanol was added to the cooled resulting mixture, which led to precipitate formation. The formed PtNP precipitates were centrifuged, washed, and dispersed in hexane. In the second reaction, synthesized PtNP was injected into a hot reacting component containing OAM, OA, and 1-octadecene under an inert condition. The reaction temperature was increased from 100 °C to 120 °C, and Fe(CO)_5_ was injected into the reacting components. The heating was further continued for 4.5 h. Upon completion of the reaction, the mixture was cooled, stirred, and precipitated. The precipitates were then dried in air (Scheme 1a) [29].

### 2.2. Preparation of Pt–Fe_3_O_4_ Hybrid Nanoparticles-Grafted Chitosan Nanocomposite Film

Chitosan was dispersed in ultra-pure water for 1 h at room temperature (RT) with continuous stirring. After 1 h, glycolic acid was injected into the reacting components and stirred for 12 h. Upon completion of the 12 h, hybrid nanoparticles were added. Next, it was stirred for 12 h at RT. The resulting mixture was then degassed for 45 min at 80 °C (Scheme 1b). Following this, the resulting mixture was cast on a glass petri dish and dried under a vacuum for 8 h at 60 °C to facilitate the dehydration of the glycolic acid-grafted chitosan polymer by forming amide bonds. The nanocomposite films were washed by the soxhlet method using methanol for the duration of 48 h to remove the unreacted glycolic acid and its oligomers. The resulting film was 0.17 mm thick. The formulations of the chitosan and the nanoparticle (Pt–Fe_3_O_4_) are given in Table 1.

### 2.3. Characterization

#### 2.3.1. Transmission Electron Microscopy

The size and morphology of the Pt–Fe_3_O_4_ hybrid nanoparticles were examined with transmission electron microscopy (HR-TEM, Model: Technai TF30, 300 kV FEG, ThermoFisher Scientific., Hillsboro, OR, USA).

#### 2.3.2. Physical Property Measuring System

The physical property measurement system (PPMS, sample vibrating magnetometer with superconducting 7T magnet, Quantum Design Inc., San Diego, CA, USA) was used to determine the magnetic property of the Pt–Fe_3_O_4_ hybrid nanoparticles. Sample holder magnetic signals were negligible, and did not have any effect on data accuracy.

#### 2.3.3. X-ray Diffraction 

The XRD pattern of the nanocomposite films was recorded using a wide angle X-ray diffraction instrument (WAXRD, Rigaku Corporation, Tokyo, Japan). The radiation used was Cu-kα at 50 kV voltage. At RT, the scan rate was 40/min with a 2θ scan range of 2° to 80°. It was used to determine the crystalline nature of the chitosan films. 

#### 2.3.4. Atomic Force Microscopy

Atomic force microscopy (AFM, Model: Nanoscope IV, Vecco Metrology, Santa Barbara, CA, USA) in contact mode was used to examine the nanocomposite films’ surface morphology. 

#### 2.3.5. FTIR Spectra

The FTIR spectra of chitosan (CS), glycolic acid (GA)-grafted chitosan (GA-g–CS), and GA-g–CS–Pt–Fe_3_O_4_ nanoparticles nanocomposite films were obtained with an attenuated total reflectance Fourier transform infrared (ATR-FTIR, Nicolet Nexus 870, Perkin Elmer Inc., Waltham, MA, USA) spectrometer with a diamond accessory (wave number range 4000–550 cm^−1^, 4 cm^−1^ resolution, 64 scans).

#### 2.3.6. Thermogravimetric Analysis

The thermogravimetric analysis (TGA, Model: TGA Q5000 instrument, TA instrument, New Castle, DE, USA) of the nanocomposite films were performed at 50 °C to 900 °C with a 20 mL/min liquid nitrogen flow rate, and a 10 °C/min heating rate. 

#### 2.3.7. Tensile Strength Testing

The Linkam TST 350 (Linkam Scientific Instruments Ltd., Waterfield, UK) was used to conduct the tensile strength testing of the films. Each nanocomposite film was cut into a dumb-bell shape and strained with a constant speed of 10 mm/min at 27 °C to break. Linkam software was used to calculate the break strain and stress. 

#### 2.3.8. Water Absorption Study

Dry and clean nanocomposite films of known weight were submerged in ultra-pure water for 24 h at 25 °C. The films were taken out from the water and dried with absorbent paper. These sample water absorption percentages were determined using Equation (1):

AB% = (W_S_ − W_D_)/W_D_ (1)

where AB% is water absorption percentage, W_S_ is the swollen film weight, and W_D_ is the dry film weight.

## 3. Results and Discussion

### 3.1. TEM Analysis of Nanoparticles

**Figure 1:**
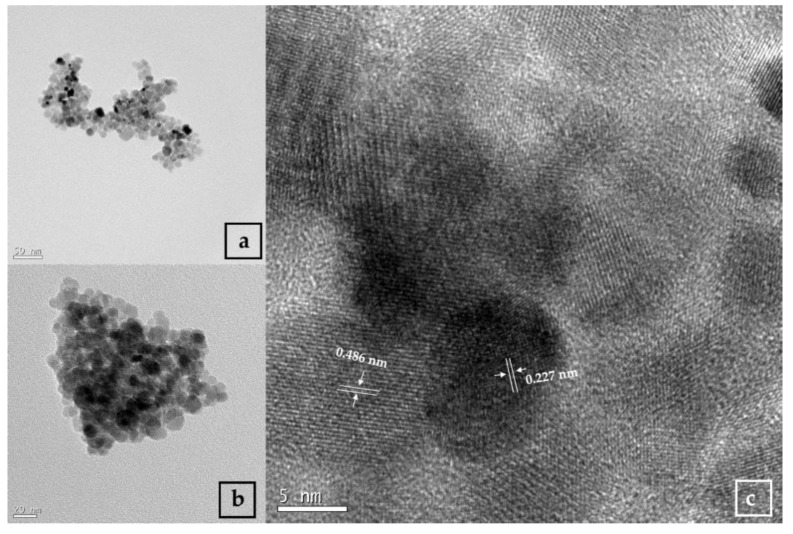
(**a**,**b**) Pt–Fe_3_O_4_ nanoparticles TEM image; (**c**) Pt–Fe_3_O_4_ nanoparticles HR-TEM image (white line indicates the gap between two lattice planes of nanoparticle).

Figure 1a,b show the TEM images of the Pt–Fe_3_O_4_ hybrid nanoparticles. It indicates that the Pt–Fe_3_O_4_ hybrid nanoparticles exhibit varying contrast, which differs from uniformly spherical or quasi-spherical to having almost the same size [30]. The high-resolution image (Figure 1c) indicates the commensurate lattice planes of the nanoparticle. The gap between adjacent lattices in the Pt domain is 0.227 nm, which matches with the value in the literature of 0.227 nm with the lattice plane value of (1 1 1) [20], and that in the Fe_3_O_4_ domain is 0.486 nm, near to the reported value of 0.488 nm for (1 1 1) faces. These nanoparticles have crystalline properties.

### 3.2. Magnetic Property Analysis of the Pt–Fe_3_O_4_ Hybrid Nanoparticles

**Figure 2:**
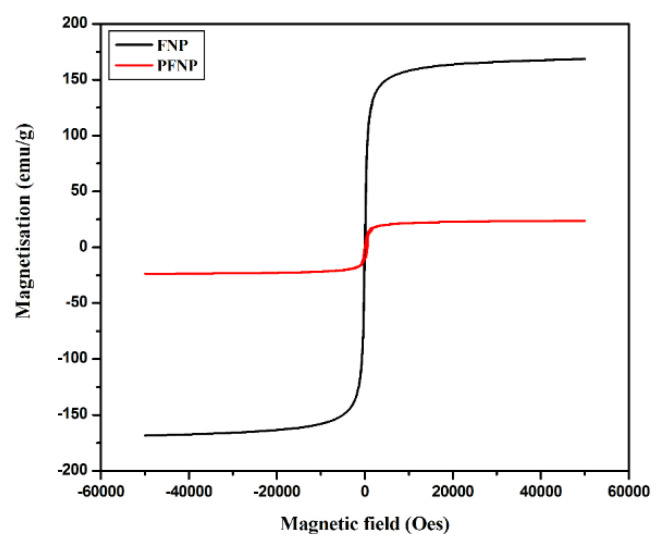
Hysteresis curve of the Pt–Fe_3_O_4_ nanoparticles (PFNP) and Fe_3_O_4_ nanoparticles (FNP).

To determine the effect of Pt (diamagnetic) on the domain of Fe_3_O_4_, it was important to investigate the Pt–Fe_3_O_4_ nanoparticle’s magnetic property (using PPMS). Figure 2 indicates the comparative hysteresis loop of Fe_3_O_4_ nanoparticles (size range 5–10 nm) with the Pt–Fe_3_O_4_ hybrid nanoparticles, which is recorded at 300 k. The hybrid nanoparticles were observed to be superparamagnetic. The saturation in magnetization is observed due to particles of Pt [31]. The shift in the magnetic property of the Pt–Fe_3_O_4_ hybrid nanoparticles confirms the linkage of Pt with Fe_3_O_4_ to form the Pt–Fe_3_O_4_ hybrid nanoparticles.

### 3.3. FTIR Analysis 

**Figure 3:**
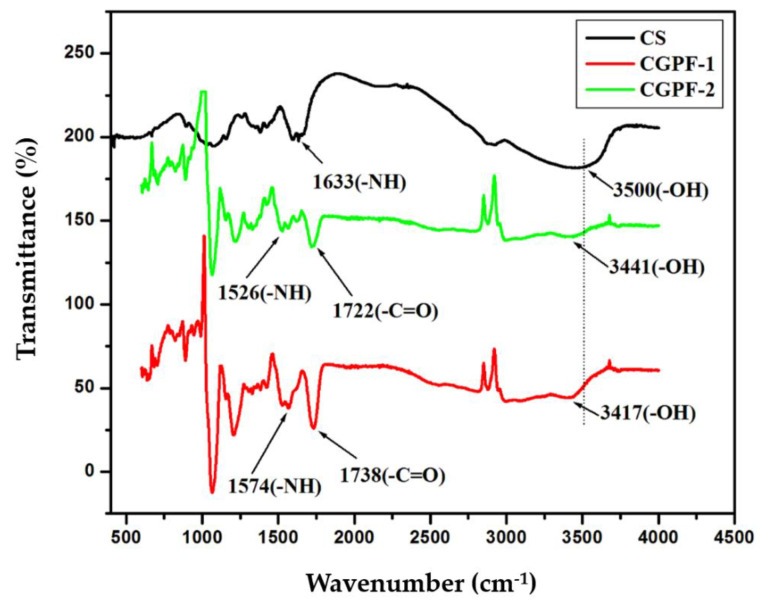
Chitosan (CS), glycolic acid-grafted–chitosan (CGPF−1) and glycolic acid-grafted–chitosan–Pt–Fe_3_O_4_ hybrid nanoparticles nanocomposite films FTIR spectra.

The CS, CGPF−1, and CGPF-2 FTIR spectra are shown in Figure 3. NH stretching (1633 cm^−1^) and -OH stretching (3500 cm^−1^) are prominent peaks in the chitosan FTIR spectrum [32]. The appearance of an additional peak at 1738 cm^−1^ corresponds to -C=O stretching. Furthermore, a shift in the -NH stretching peak to 1574 cm^−1^ is observed in the CGPF−1 spectrum, which confirms the GA interaction with the chitosan -NH_2_ functional group. The –NH-C=O amide linkage formation between the CS and the GA indicates grafting on chitosan. The Pt–Fe_3_O_4_ nanoparticles interact with the glycolic acid –C=O functional group and the chitosan –OH functional group through chemical bonds, which can cause a shift in the peak of -C=O stretching (1722 cm^−1^) and -OH stretching (3417 cm^−1^) in the CGPF-2 spectrum.

### 3.4. XRD Analysis 

**Figure 4:**
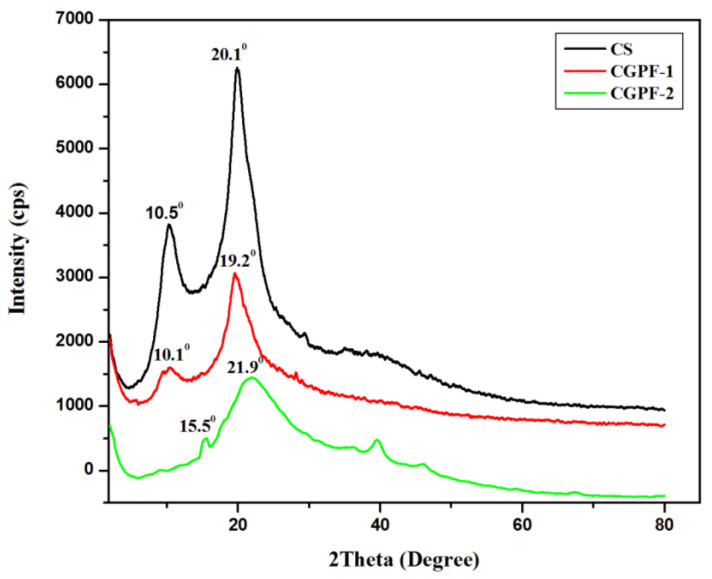
X-ray diffraction spectra of chitosan, grafted chitosan, and grafted chitosan–Pt–Fe_3_O_4_ nanoparticles nanocomposite films.

Figure 4 shows XRD peaks of the CS, CGPF−1, and GA-g–CS–Pt–Fe_3_O_4_ hybrid nanoparticles nanocomposite (CGPF-2) films. The CS shows two distinct crystalline peaks with 2θ values 10.5°and 20.1° [33]. These peaks correspond to the long range order and structural sequences of chitosan. Chitosan’s structure is highly influenced by its processing methods, such as the drying method, precipitation method, and dissolving method. It also depends upon its molecular weight and deacetylation level [34]. In the grafted chitosan (CGPF−1) film’s XRD, the peaks have been moved from 10.5° to 10.1°, and 20.1° to 19.2°, confirming that the glycolic acid has grafted onto the chitosan chains. The XRD pattern of the GA-g–CS–Pt–Fe_3_O_4_ nanoparticles nanocomposite (CGPF-2) film shows a shift in the peaks from 2θ = 10.1° to 15.5° and, 19.2° to 21.9°. The shift in peaks of the GA-g–CS–Pt–Fe_3_O_4_ nanoparticles nanocomposite film shows the interaction of the nanoparticles with the grafted chitosan matrix. The broadening of the peak at 21.9° suggests that nanoparticles incorporation in the grafted chitosan polymer matrix lowers the crystallinity of the polymer film [35]. A small peak at 40 ^0^ corresponds to the Pt–Fe_3_O_4_ hybrid nanoparticles.

### 3.5. Morphological Studies 

**Figure 5:**
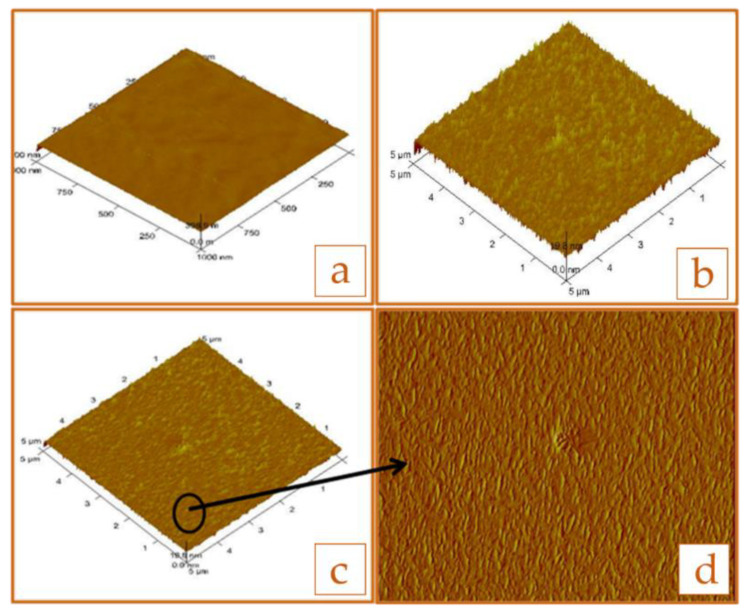
(**a**) AFM image of the pure chitosan film; (**b**) AFM image of the grafted chitosan film. (**c**,**d**) AFM image of the grafted chitosan–Pt–Fe_3_O_4_ hybrid nanoparticles nanocomposite film.

The surface topography of the pure chitosan, the grafted chitosan, and the GA-g–CS–Pt–Fe_3_O_4_ nanoparticles nanocomposite films are illustrated with AFM. The AFM image size range is 5 μm × 5 μm. Figure 5a shows the AFM image of the chitosan film with a smooth surface. Upon grafting the chitosan, the roughness and height of the film’s surface is also increased (Figure 5b). The AFM image of the GA-g–CS–Pt–Fe_3_O_4_ nanoparticles nanocomposite film depicts the integration of nanoparticles into the chitosan matrix (Figure 5c,d).

### 3.6. Water Absorption Studies

The biopolymer chitosan is hydrophilic in nature, but it does not absorb water very efficiently. The lower water absorption is due to the intramolecular and intermolecular hydrogen bond formation in the molecular structure of the chitosan due to its hydroxyl and amino functional groups. The grafting of chitosan increases its water absorption properties. Grafting of chitosan leads to a breakage in molecular integrity, which results in the exposure of its functional groups in water. The grafted chitosan film swells in water. Its swelling degree depends upon the grafting potential of polymer, osmotic pressure, and degree of ionization [36]. Singh et al. [36] reported the synthesis of lactic acid-grafted chitosan–montmorillonite (MMT) clay nanocomposite films. They observed that upon grafting the chitosan with lactic acid, increased the water absorption property of the chitosan film. The decrease in water absorption of the lactic acid-grafted chitosan—montmorillonite (MMT) clay nanocomposite films were observed with the increase in content of montmorillonite clay in grafted chitosan polymer.

In the present work, the water absorption of the GA-g–CS–Pt–Fe_3_O_4_ nanoparticles nanocomposite film decreases with the raising concentration of Pt–Fe_3_O_4_ nanoparticles in the grafted chitosan polymer. It is possibly due to the linkage of nanoparticles with the polymer. This results in crosslinking point formation, which acts as a barrier to restrict water penetration in the chitosan matrix. Since nanoparticles are hydrophobic, resulting nanocomposites are expected to be hydrophobic. The formation of nanocomposites occurs through the chemical bond formation between the Pt–Fe_3_O_4_ nanoparticles and the polymer, resulting in a reduction in water retention. The results of the water retention analysis of the GA-g–CS–Pt–Fe_3_O_4_ nanoparticles nanocomposite films with standard deviation (SD) are shown in Table 2.

The nanocomposite films absorb water up to 24 h until equilibrium is achieved. The swollen films were dried under a vacuum at 65 °C. This determined the capacity of the nanocomposite film to absorb water. The results indicate that nanocomposite films can retain the water for a longer duration. The grafted chitosan indicated a rise in the capacity for water retention. Upon increasing the nanoparticle content, the water retention decreases, and the drying time of the film up to constant weight increases. Therefore, nanoparticles act as a physical barrier that prevents moisture from escaping the film.

### 3.7. Tensile Strength Testing 

**Figure 6:**
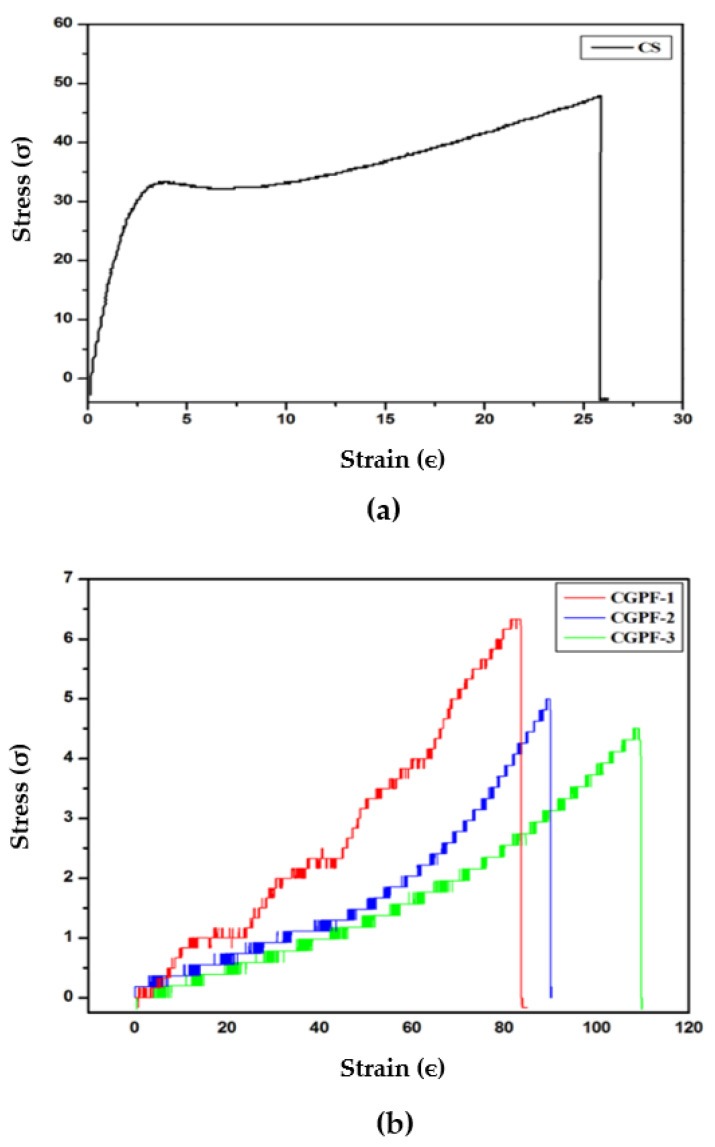
(**a**) Stress–strain curve of pure chitosan film. (**b**) Stress–strain curve of grafted chitosan film and GA-g–CS–Pt–Fe_3_O_4_ nanoparticles nanocomposite films.

The mechanical properties of the chitosan were unpredictable and lacking the accuracy in the mode of study in regard to crosshead speed or molecular weight [28,37,38,39]. As a result, the chitosan’s tensile properties were first investigated (Figure 6a). Each of the nanocomposite films had a uniform thickness of 0.17 mm and were semi-transparent with a strain of standard deviation value ± 1%. The pure chitosan film exhibited a break at the stress of 47.97 MPa and strain of 25.83 ± 1%. The tensile properties changed drastically with the application of crosshead speed. The grafted chitosan exhibited an increase in the elasticity of film with a relatively high break at strain 83.29 ± 1%. The incorporation of nanoparticles in the grafted chitosan matrix further increased the elasticity of the film, with higher break at strain values of 89.76 ± 1% and 109.20 ± 1% (Table 3). This shows that nanoparticles improved the elastic property of the polymer film (Figure 6b). The molecular structure of the chitosan involves several amino (–NH2) and (–OH) functional groups. These functional groups favor intramolecular hydrogen bonding and restrict the chitosan chain movement, which provides mechanical strength to the chitosan film. The incorporation of Pt–Fe_3_O_4_ hybrid nanoparticles in the chitosan polymer prevents intramolecular hydrogen bonding and leads to the formation of intermolecular bonds. These intermolecular bonds allow the chitosan chain to rotate. This rotation of the chitosan chain results in an increase in the tensile properties of the nanocomposite films [40]. 

### 3.8. Thermogravimetric Analysis 

**Figure 7:**
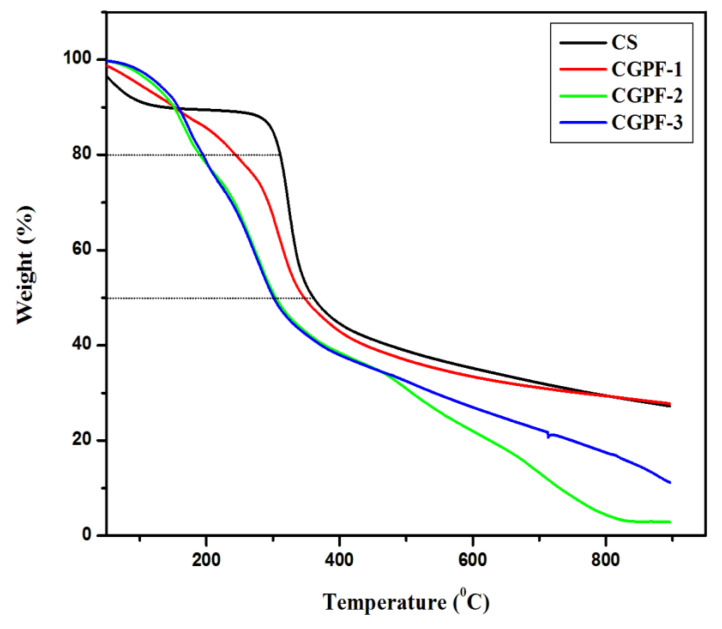
Thermogravimetric curves of nanocomposite films.

Figure 7 shows the thermogravimetric analysis of the pure chitosan polymer film and the grafted chitosan–hybrid nanoparticles-based nanocomposite films. The temperature of thermal decomposition at 20% and 50% weight loss was determined under nitrogen flow. Along with that, the yield of charred residue was also determined. This indicated two steps of non-oxidative degradation. The weight loss at 50−150 °C is mainly associated with water absorbed by the chitosan. It is indicated that degradation and deacetylation of chitosan causes weight loss in the temperature range of 200–350 °C [41]. The temperature of the thermal degradation of chitosan reduced by 20–30 °C after grafting; however, a decrease of 50–60 °C was observed upon addition of the nanoparticles to the polymer matrix (Table 4). The highest char residues (27.7% at 900 °C) were observed for the grafted chitosan film. It is interesting to note that increases in char residue were observed upon increasing the nanoparticles’ wt %. This result indicates the significant effect of grafted glycolic acid and incorporated nanoparticles on chitosan’s thermal properties. 

## 4. Conclusions

In this study, novel organic–inorganic nanocomposites were synthesized using the chitosan polymer and hybrid nanoparticles. The interaction of chitosan, a cationic polymer, with Pt–Fe_3_O_4_ nanoparticles is through the chemical bond, which results in an enhancement in the functional and structural properties. The chitosan grafting with glycolic acid induces a water retention property in the chitosan. The glycolic acid-grafted chains act as a plasticizer, which provides flexibility to polymer films. An increased concentration of nanoparticles in the polymer matrix increased the elasticity of the film. A decrease in water absorption is observed when increasing the content of the nanoparticles, which induces a branched crystalline structure in the grafted nanocomposite films. Properties of water retention and elastic strength were explored that could be applied to a variety of biomedical areas, including drug delivery and tissue engineering.

## Figures and Tables

**Table 1:** The formulation of glycolic acid-grafted chitosan with hybrid nanoparticles.

Sample Code	Chitosan (g)	Glycolic Acid (g)	Nanoparticle (mg)
CS	1	0	0
CGPF-1	1	1	0
CGPF-2	1	1	20
CGPF-3	1	1	40

**Table 2:** Water retention behavior of nanocomposite films.

Sample Code	Water Absorption (%) with SD
CS	54 ± 0.5
CGPF−1	77.7 ± 0.5
CGPF-2	62 ± 0.5
CGPF-3	33.2 ± 0.5

**Table 3:** Tensile strength and testing of the chitosan and nanocomposites.

Sample Code	Break Stress (σ)	Break Strain (ε) with SD (%)
CS	47.97	25.83 ± 1
CGPF−1	6.38	83.29 ± 1
CGPF-2	4.99	89.76 ± 1
CGPF-3	4.50	109.20 ± 1

**Table 4:** Thermogravimetric analysis of chitosan and its nanocomposite films.

Sample Code	Temperature at 20% Loss °C	Temperature at 50% Loss °C	Char 900 °C (wt %)
CS	310	360	27.1
CGPF−1	246	347	27.7
CGPF-2	188	305	2.91
CGPF-3	197	300	11.14

## Data Availability

The data is only available in the author Ph.D thesis.
